# Efficacy and Safety of Biomaterials and Antimicrobial Dressings in the Treatment of Diabetic Foot Ulcers: A Systematic Review and Network Meta-Analysis

**DOI:** 10.1155/jdr/1548984

**Published:** 2025-10-08

**Authors:** Jing Zhang, Yanan Zhao, Ziwei Hu, Qi Qi, Mingyue Jia, Zexinyao Ren, Jiaqi Dai, Liwei Jing

**Affiliations:** School of Nursing, Capital Medical University, Beijing, China

**Keywords:** antimicrobial dressings, biomaterials, diabetic foot ulcers, efficacy evaluation, network meta-analysis, randomized controlled trial

## Abstract

This systematic review and network meta-analysis is aimed at comparing the efficacy and safety of various wound dressings in the treatment of diabetic foot ulcers, as differences in outcomes among these materials remain unclear. A comprehensive literature search was conducted across major Chinese and international databases up to October 2024, identifying 35 randomized controlled trials involving 2631 patients. Data were extracted and quality-assessed using standardized methods, and a network meta-analysis was performed using appropriate statistical software. The results showed that, compared with traditional dressings, novel biomaterials (e.g., growth factors, amniotic membrane, platelet-rich plasma, and hydrogels) and antimicrobial dressings (e.g., silver ion dressings) combined with basic fibroblast growth factor or hydrogel significantly shortened wound healing time. In terms of healing efficiency, combinations involving epidermal growth factor, amniotic membrane, or platelet-rich plasma with hydrogel were significantly more effective than traditional dressings. The surface under the cumulative ranking curve values indicated that the most effective interventions were antimicrobial dressings (silver ion) combined with basic fibroblast growth factor for wound healing time and epidermal growth factor–based regimens for healing efficiency. Importantly, sensitivity analyses excluding studies at high risk of bias (allocation/blinding) demonstrated that the ranking for ulcer healing time was unstable—attenuated for platelet-rich plasma but more favorable for honey dressings—whereas estimates for healing efficiency remained robust. These findings suggest that conclusions regarding healing time are particularly sensitive to study quality and should be interpreted with caution. No serious adverse events were reported, and most interventions were well tolerated. These findings suggest that biomaterial and antimicrobial dressings, particularly when used in combination with traditional methods, offer clear advantages in the management of diabetic foot ulcers and should be prioritized in clinical practice.

**Trial Registration:** CRD42024578762

## 1. Introduction

Diabetic foot ulcers (DFUs), a severe complication of diabetes mellitus, are characterized by skin ulceration and/or deep tissue destruction in the distal lower extremities, resulting from diabetic neuropathy and/or varying degrees of peripheral vascular disease, with or without infection [1]. Globally, approximately 537 million individuals are affected by diabetes [2], among whom 19%–34% are at risk of developing DFUs [3]. This complication not only accounts for approximately 20% of lower limb amputations but is also associated with a 10% all-cause mortality rate within 1 year of initial diagnosis [4]. Beyond the profound impact on individual health, DFUs impose a significant socioeconomic burden. In the United States alone, the annual direct costs of diabetes care are estimated at 273 billion, with direct costs reaching 90 billion, of which foot complications constitute a major expenditure [5]. Given these substantial implications, the prevention and effective management of DFUs are of paramount importance.

Local wound management is a critical component of DFU care. In clinical practice, wound dressings play a pivotal role by providing an optimal healing environment, preventing infection, and managing exudate. Both the International Working Group on the Diabetic Foot (IWGDF) [6] and the Chinese Diabetes Society [7] recommend the use of appropriate dressings to control exudate and infection, maintain a moist wound environment, and promote ulcer healing. Compared to traditional dressings, biomaterials have garnered significant attention due to their ability to enhance keratinocyte migration, collagen formation, angiogenesis, and scar reduction in both acute and chronic wounds [8]. In contrast, antimicrobial dressings, such as those incorporating silver ions, primarily function to reduce bacterial load and are classified separately as per the European Medicines Agency (EMA) and ASTM International standards [9, 10]. However, the comparative efficacy and safety profiles of various biomaterials and antimicrobial dressings remain underexplored, necessitating further evaluation.

Network meta-analysis (NMA) offers a robust statistical framework for simultaneously comparing multiple interventions, even in the absence of direct comparative trials. Given the diversity of available biomaterials and antimicrobial dressings and their distinct mechanisms of action, this methodology enables a more precise assessment of their relative efficacy. This study is aimed at systematically reviewing and conducting a NMA to evaluate the efficacy and safety of different biomaterials and antimicrobial dressings in the treatment of DFUs. The findings are expected to inform clinical decision-making by identifying the safest and most effective options, ultimately improving patient outcomes and alleviating the disease burden.

## 2. Research Design and Methods

This study strictly adhered to the Preferred Reporting Items for Systematic Reviews and Meta-Analyses (PRISMA) guidelines and has been registered with the International Prospective Register of Systematic Reviews (PROSPERO, https://www.crd.york.ac.uk/PROSPERO).

## 3. Literature Search

Chinese databases, including China National Knowledge Infrastructure (CNKI), VIP, Wanfang Data, and China Biology Medicine Database (CBM), were searched. English databases, including PubMed, Embase, Cochrane Library, and Web of Science, were also searched. The search terms were as follows: “diabetic foot ulcer, DFU, foot ulcer, platelet-rich plasma (PRP), silver ions, bandage, dressing, randomised”. The search period ranged from the inception of each database to October 2024. The detailed literature search strategy is shown in Table 1 and Figure S1.

### 3.1. Inclusion Criteria

• Study type: Only randomized controlled trials (RCTs) were included.
• Study population: Patients diagnosed with DFUs, with clear diagnostic criteria described in the study.
• Interventions: The control group received traditional dressings (e.g., saline gauze, Vaseline gauze, or antibacterial gauze) or routine debridement; the intervention group received additional treatments such as biomaterials (e.g., growth factors, PRP, amniotic membrane, hydrogel, and honey dressings) or antimicrobial dressings (e.g., silver ion dressings) in combination with the control group's treatment.
• Outcome measures: Primary outcomes included wound healing time and healing efficiency of DFUs.

### 3.2. Exclusion Criteria

Studies such as dissertations, conference abstracts, review articles, and animal experiments were excluded. Studies with unavailable or incomplete raw data were also excluded. For duplicate publications, only the most recent version was included.

## 4. Literature Screening and Data Extraction

The retrieved literature was imported into the NoteExpress software. Two researchers independently reviewed the titles and abstracts of the studies, removed duplicates, and then conducted full-text screening based on the inclusion and exclusion criteria to exclude studies that did not meet the requirements. Subsequently, the included studies were cross-checked, and any discrepancies were resolved through discussion or consultation with a third-party expert. Data extraction included the following information: publication year, first author, sample size, age, gender, interventions, outcome measures, and disease duration.

### 4.1. Quality Assessment of Included Studies

Two researchers independently assessed the quality of the included studies using the Cochrane Risk of Bias Tool [11] and RevMan 5.4 software recommended by the Cochrane Collaboration. The quality assessment covered six domains: (1) random sequence generation, (2) allocation concealment, (3) blinding, (4) completeness of outcome data, (5) selective reporting, and (6) other potential sources of bias. Based on the evaluation criteria, the risk of bias for each study was categorized as high, moderate, or low. Any discrepancies were resolved through discussion or consultation with a third-party expert.

### 4.2. Statistical Analysis

Wound healing time, as a continuous variable, was expressed using the standardized mean difference (SMD) and its 95% confidence interval (CI). The overall effectiveness rate, as a binary variable, was expressed using the odds ratio (OR) and its 95% CI. RevMan 5.4 software (https://www.cochranelibrary.com) was used for bias assessment and traditional meta-analysis, with heterogeneity evaluated using the *I*^2^ statistic. When *I*^2^ < 50% and *p* ≥ 0.05, heterogeneity among the included studies was considered insignificant, and a fixed-effects model was applied. When *I*^2^ > 50% and *p* < 0.05, significant heterogeneity was indicated, and sensitivity or subgroup analyses were conducted to explore its sources. If heterogeneity could not be reduced, a random-effects model was used.

NMA was performed using Stata 18.0 software (https://www.stata.com): (1) A network evidence graph was plotted to illustrate the quantitative relationships among interventions. (2) If closed loops were present in the network graph, global and local inconsistency tests were performed. If all comparison results showed *p* > 0.05, consistency was considered good, and direct and indirect comparisons were deemed consistent. Loop inconsistency tests were then conducted to evaluate the consistency of closed loops for each outcome. If the 95% CI of the loop inconsistency factor (IF) included 0, direct and indirect evidence was considered consistent, and a consistency model was applied; otherwise, an inconsistency model was used. (3) Cumulative probability plots (SUCRA) were generated to rank the efficacy of different treatments. (4) A comparison-adjusted funnel plot was created using Stata 18.0 software to identify publication bias and small-study effects in the intervention network. We assessed publication bias in the studies utilizing both the funnel plot and Egger's test. An asymmetric distribution in the funnel plot indicates the potential presence of publication bias or small-study effects. In these instances, publication bias was deemed present if Egger's test yielded a *p* value of less than 0.05. A sensitivity analysis was conducted using the trim-and-fill analysis. If the pooled effect size and *p* value exhibited no significant changes in magnitude or direction before and after the trimming and filling process, the results were considered robust. The significance level was set at *α* = 0.05.

## 5. Results

### 5.1. Results of Literature Inclusion

**Figure 1:**
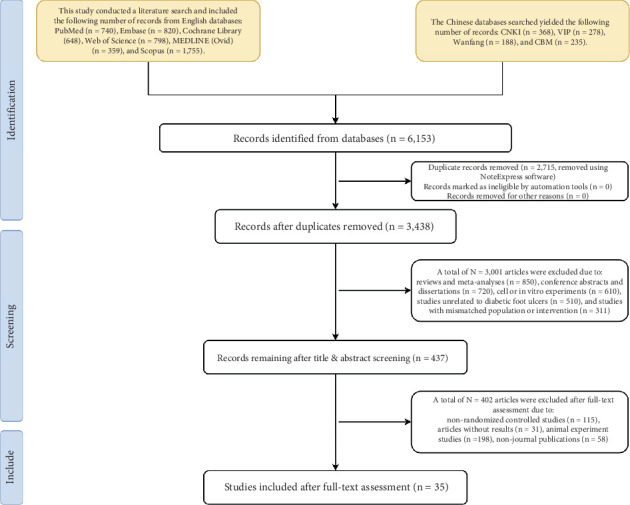
Flowchart of literature screening.

A total of 6153 articles were initially identified through database searches. After removing 2715 duplicate references, 3438 articles remained for title and abstract screening. During this step, 3403 articles were excluded for the following reasons: 115 due to nonrandomized controlled trial (non-RCT) design and 3001 due to irrelevant study content—including reviews and meta-analyses (*n* = 850), conference abstracts and dissertations (*n* = 720), cell or in vitro experiments (*n* = 610), studies unrelated to DFUs (*n* = 510), and studies with mismatched population or intervention (*n* = 311). Additionally, 31 articles without results were excluded. Following full-text review, 198 animal experiment studies and 58 nonjournal publications were further excluded. Ultimately, 35 articles met the inclusion criteria and were included in the analysis. The literature screening process is illustrated in Table S1 and Figure 1.

### 5.2. Basic Characteristics of Included Studies

This study included a total of 35 RCTs [12–46], involving 2429 patients (1181 in the control group and 1248 in the experimental group). Among these, one study was a four-arm trial [45], while the remaining 34 were two-arm studies. The basic characteristics of the included studies are detailed in Table 2.

### 5.3. Included Study Quality Assessment

**Figure 2:**
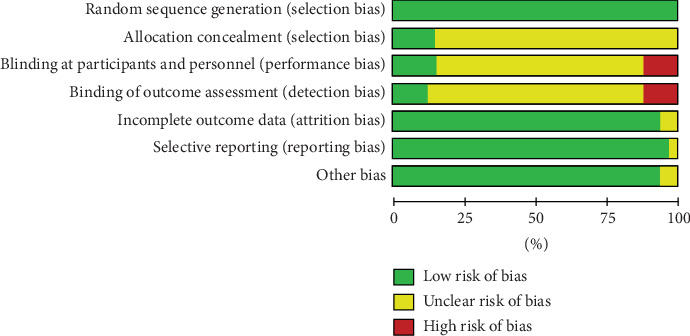
Risk of bias graph.

**Figure 3:**
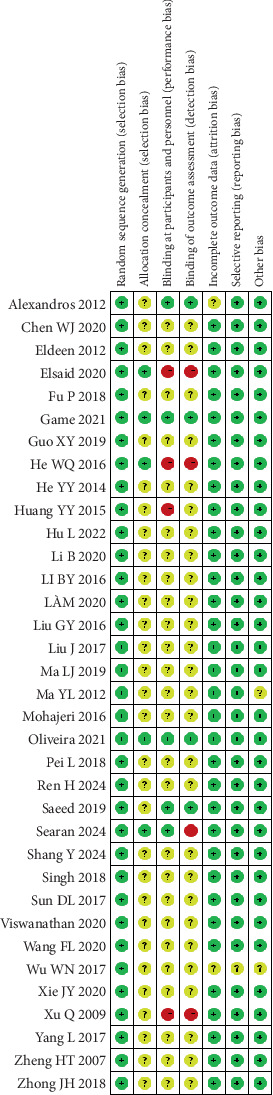
Risk of bias summary.

All 35 included studies mentioned random allocation. Among these, 19 studies [17, 19, 20, 22–24, 28, 29, 32, 33, 35, 37, 38, 40–45] reported specific methods for generating random sequences, primarily using random number tables and computer software. Five studies [19, 40–42, 45] detailed the methods of allocation concealment. Nine studies [13, 16, 18, 19, 31, 40–42, 45] reported the use of blinding, with four of these being double-blind trials [16, 31, 41, 42]. None of the included studies had issues with selective reporting or missing outcomes, and no other risks of bias were mentioned in the texts. The risk of bias graph and summary graph for the included literature are shown in Figures 2 and 3.

### 5.4. Traditional Meta-Analysis Results

**Figure 4:**
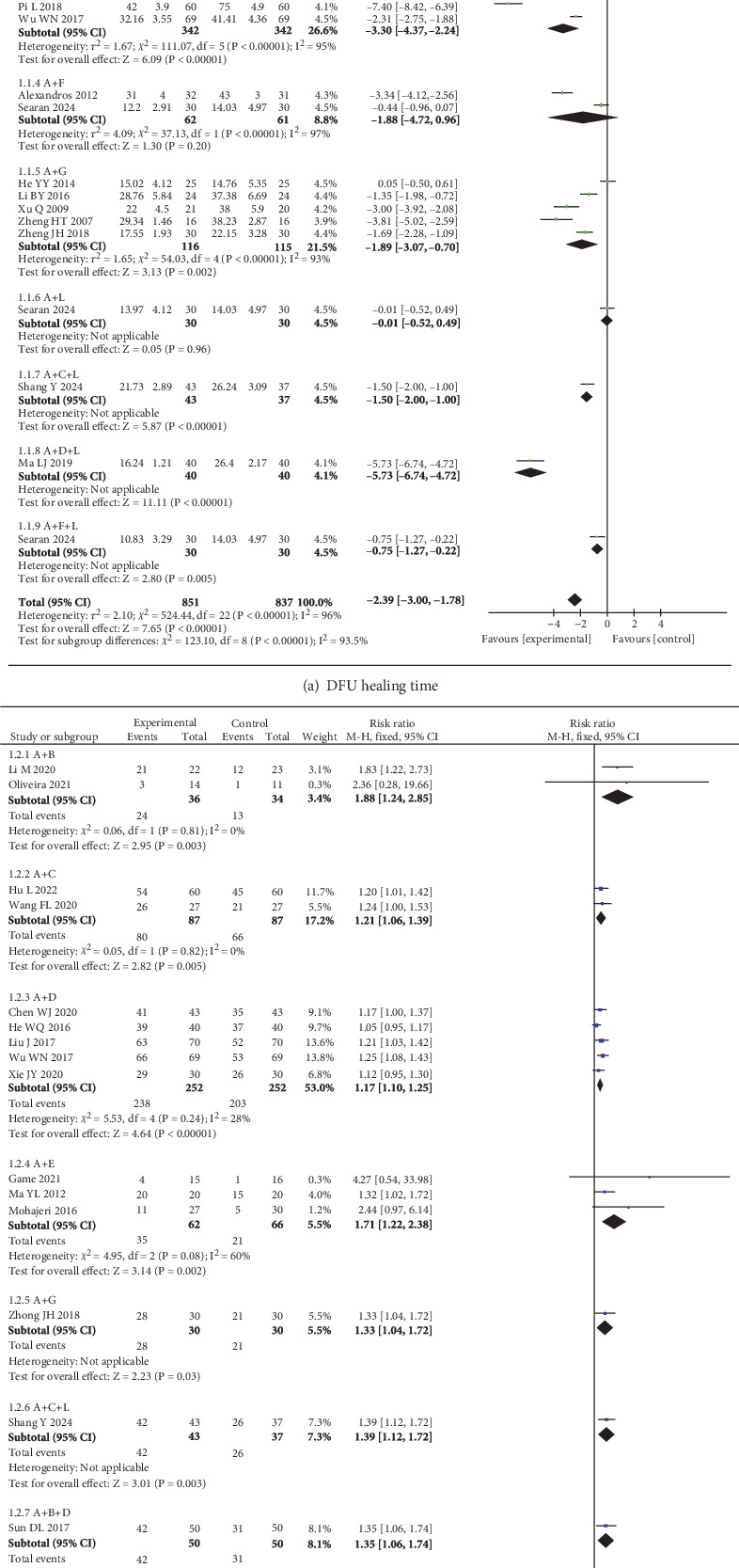
Forest plots for outcome-specific subgroups in the meta-analysis. Note: (A) Traditional dressings (oil sand, cotton cloth, dry gauze, and bandages). (B) Epidermal growth factor. (C) Platelet-rich plasma gel (APG). (D) Antimicrobial dressings (silver ion–based). (E) Biomaterials (amniotic membrane). (F) Honey dressings. (G) Basic fibroblast growth factor. (H) Hydrogel.

Regarding the improvement of ulcer healing time, a total of 21 studies [12, 13, 16–19, 21, 24–27, 29, 30, 32, 37–40, 43, 45, 46] involving 9 wound dressings were included. The overall heterogeneity test showed *I*^2^ = 96%, *p* < 0.05, indicating significant heterogeneity among the studies. After excluding studies with high heterogeneity [29, 30], the heterogeneity remained high (*I*^2^ = 94%, *p* < 0.05). Therefore, only descriptive analysis was performed. Compared to the control group (routine debridement or traditional dressings), the experimental group (SMD = −2.39, 95% CI: −3.00, −1.78) significantly reduced the wound healing time. Subgroup analysis based on different intervention methods revealed that traditional dressings combined with epidermal growth factor (EGF), APG, silver ion dressings, honey dressings, basic fibroblast growth factor (bFGF), APG + hydrogel, silver ion + hydrogel, and honey dressings + hydrogel significantly shortened wound healing time compared to traditional dressings alone (*p* < 0.05), as shown in Figure 4a.

In terms of ulcer healing efficiency, 15 studies [14, 19, 22, 23, 25–27, 34, 35, 37, 38, 41–43, 46] involving 7 wound dressings were included. The overall heterogeneity test showed *I*^2^ = 46%, *p* < 0.05, indicating low heterogeneity among the studies. Therefore, a fixed-effects model was used for analysis. The results demonstrated that the experimental group (RR = 1.27, 95% CI: 1.20, 1.35) significantly improved the effective ulcer healing rate compared to the control group. Subgroup analysis based on specific intervention methods showed that all wound dressings significantly improved the effective ulcer healing rate compared to traditional dressings alone (*p* < 0.05), as shown in Figure 4b.

### 5.5. The Results of NMA

#### 5.5.1. Outcome Indicator Evidence Network

**Figure 5:**
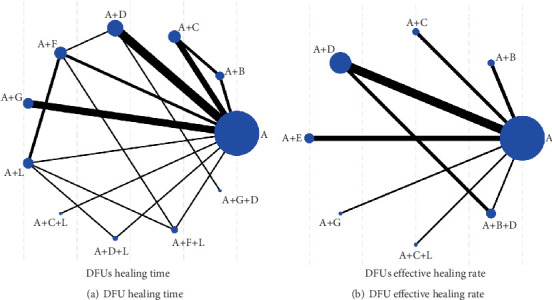
Network diagram of evidence.

Among the 35 included studies, 27 reported the healing time of DFU [12, 13, 15–21, 24–32, 34, 37–40, 43–46]. Eleven interventions formed a network graph centered on routine debridement or traditional dressings, comprising six triangular closed loops: (1) routine debridement or traditional dressings − routine debridement or traditional dressings + EGF − routine debridement or traditional dressings + PRP; (2) routine debridement or traditional dressings − routine debridement or traditional dressings + silver ion dressings − routine debridement or traditional dressings + honey dressings; (3) routine debridement or traditional dressings − routine debridement or traditional dressings + honey dressings − routine debridement or traditional dressings + hydrogel; (4) routine debridement or traditional dressings − routine debridement or traditional dressings + honey dressings − routine debridement or traditional dressings + honey dressings + hydrogel; (5) routine debridement or traditional dressings − routine debridement or traditional dressings + hydrogel − routine debridement or traditional dressings + silver ion dressings + hydrogel; (6) routine debridement or traditional dressings − routine debridement or traditional dressings + hydrogel − routine debridement or traditional dressings + honey dressings + hydrogel (Figure 5a).

Additionally, among the 35 included studies, 17 reported the effective rate of DFU [14, 19, 22, 23, 25–27, 33–38, 41–43, 46]. Eight interventions formed a network graph centered on routine debridement or traditional dressings, comprising one triangular closed loop:

Routine debridement or traditional dressings − routine debridement or traditional dressings + silver ion dressings − routine debridement or traditional dressings + silver ion dressings + EGF (Figure 5b).

The size of the nodes represents the number of interventions, and the thickness of the lines represents the number of studies comparing the interventions.

#### 5.5.2. Inconsistency Test Results

For the outcome of ulcer healing time, the global inconsistency test returned a *p* value of 0.470. To further evaluate local inconsistency, the node-splitting method was applied, revealing that all pairwise comparisons yielded *p* values exceeding 0.05. Similarly, for ulcer healing efficiency, the global inconsistency test produced a *p* value of 0.9351, while the node-splitting analysis confirmed no significant local inconsistency, as all pairwise comparisons also had *p* values greater than 0.05. Moreover, the 95% CI of the IF encompassed zero for both outcomes.

#### 5.5.3. NMA Under the Consistency Model

##### 5.5.3.1. Ulcer Healing Time

**Figure 6:**
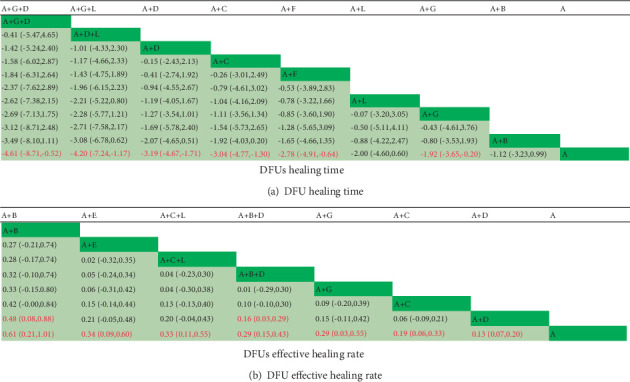
Results of network meta-analysis for different wound dressings in DFU treatment. Note: (A) Traditional dressings (oil sand, cotton cloth, dry gauze, and bandages). (B) Epidermal growth factor. (C) Platelet-rich plasma gel (APG). (D) Antimicrobial dressings (silver ion–based). (E) Biomaterials (amniotic membrane). (F) Honey dressings. (G) Basic fibroblast growth factor. (H) Hydrogel.

The following interventions demonstrated a significant reduction in ulcer healing time compared to traditional dressings alone (*p* < 0.05): traditional dressings combined with silver ion dressings and bFGF, traditional dressings combined with silver ion dressings and hydrogel, traditional dressings combined with silver ion dressings, traditional dressings combined with PRP, traditional dressings combined with honey dressings, and traditional dressings combined with bFGF. No significant differences were observed among other intervention comparisons (*p* > 0.05) (Figure 6a).

##### 5.5.3.2. Healing Efficiency

Traditional dressings combined with EGF and traditional dressings combined with EGF and silver ion dressings showed significantly higher healing efficiency compared to traditional dressings combined with silver ion dressings alone (*p* < 0.05). Furthermore, the following interventions were significantly more effective than traditional dressings alone (*p* < 0.05): traditional dressings combined with EGF, traditional dressings combined with amniotic membrane, traditional dressings combined with PRP and hydrogel, traditional dressings combined with EGF and silver ion dressings, traditional dressings combined with bFGF, traditional dressings combined with PRP, and traditional dressings combined with silver ion dressings. No significant differences were observed among other intervention comparisons (*p* > 0.05) (Figure 6b).

#### 5.5.4. Cumulative Probability Ranking Results of Included Studies

##### 5.5.4.1. Ulcer Healing Time

**Figure 7:**
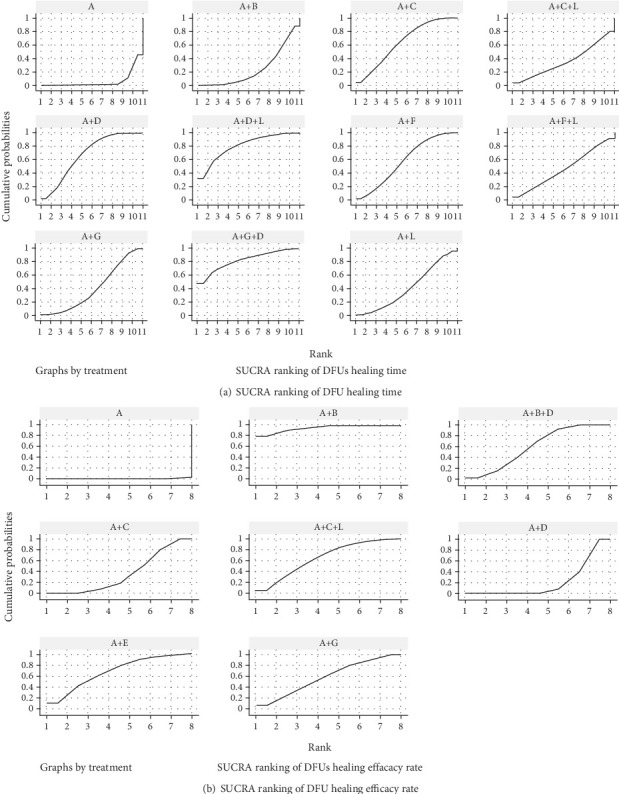
SUCRA ranking plot. Note: (A) Traditional dressings (oil sand, cotton cloth, dry gauze, and bandages). (B) Epidermal growth factor. (C) Platelet-rich plasma gel (APG). (D) Antimicrobial dressings (silver ion–based). (E) Biomaterials (amniotic membrane). (F) Honey dressings. (G) Basic fibroblast growth factor. (H) Hydrogel.

The ranking of wound dressings based on SUCRA values was as follows: *A* + *G* + *D* (82.2%) > *A* + *D* + *L* (81.1%) > *A* + *D* (68.0%) > *A* + *C* (64.4%) > *A* + *F* (58.2%) > *A* + *F* + *L* (47.2%) > *A* + *L* (41.2%) > *A* + *G* (40.6%) > *A* + *C* + *L* (36.4%) > *A* + *B* (24.9%) > *A* (5.8%) (Figure 7a).

##### 5.5.4.2. Healing Efficiency

The ranking of wound dressings based on SUCRA values was as follows: *A* + *B* (93.5%) > *A* + *E* (67.2%) > *A* + *C* + *L* (65.8%) > *A* + *B* + *D* (59.2%) > *A* + *G* (56.7%) > *A* + *C* (36.3%) > *A* + *D* (21.0%) > *A* (0.3%) (Figure 7b).

### 5.6. Sensitivity Analyses Excluding High-Risk Studies

To assess the robustness of our findings, we performed sensitivity analyses by excluding studies judged to be at high risk of bias in allocation concealment or blinding. The results indicated that the overall network remained consistent, with no significant global or local inconsistency detected. While the ranking of some interventions (e.g., PRP gel and honey dressings) changed, silver-based dressings consistently ranked among the top interventions, underscoring the stability of the main conclusions. Detailed results and SUCRA ranking plots are provided in Figures S1 and S2.

### 5.7. Publication Bias Analysis

**Figure 8:**
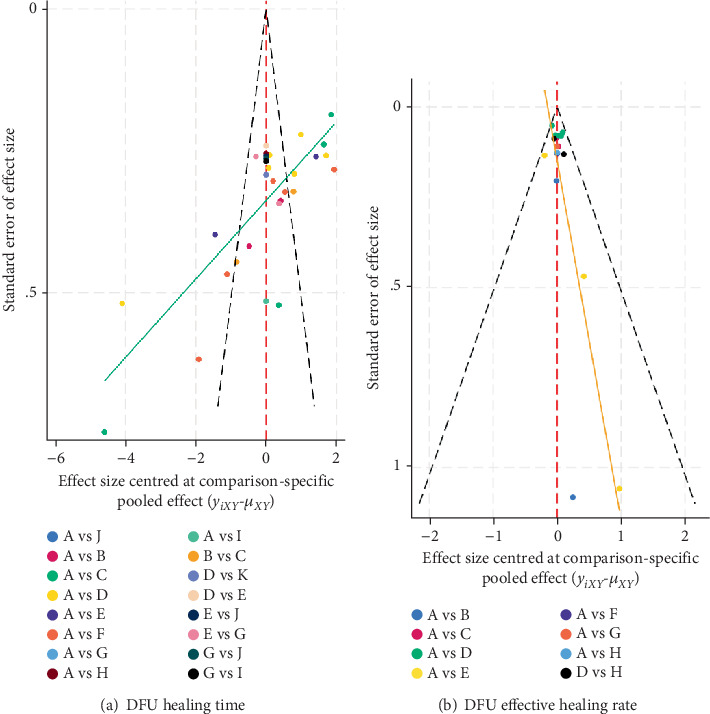
Comparison-adjusted funnel plot. Note: (A) Traditional dressings (oil sand, cotton cloth, dry gauze, and bandages). (B) Epidermal growth factor. (C) Platelet-rich plasma gel (APG). (D) Antimicrobial dressings (silver ion–based). (E) Biomaterials (amniotic membrane). (F) Honey dressings. (G) Basic fibroblast growth factor. (H) Hydrogel.

Funnel plots were constructed to assess publication bias for ulcer healing time and healing efficiency. As shown in Figure 8a, the funnel plot for ulcer healing time was asymmetrical, with some data points located outside the funnel, suggesting potential small-study effects and publication bias. Egger's test further confirmed potential bias (*p* = 0.006). In contrast, for healing efficiency, although the funnel plot (Figure 8b) appeared largely symmetrical, Egger's test indicated small-study effects (*p* < 0.001).

**Figure 9:**
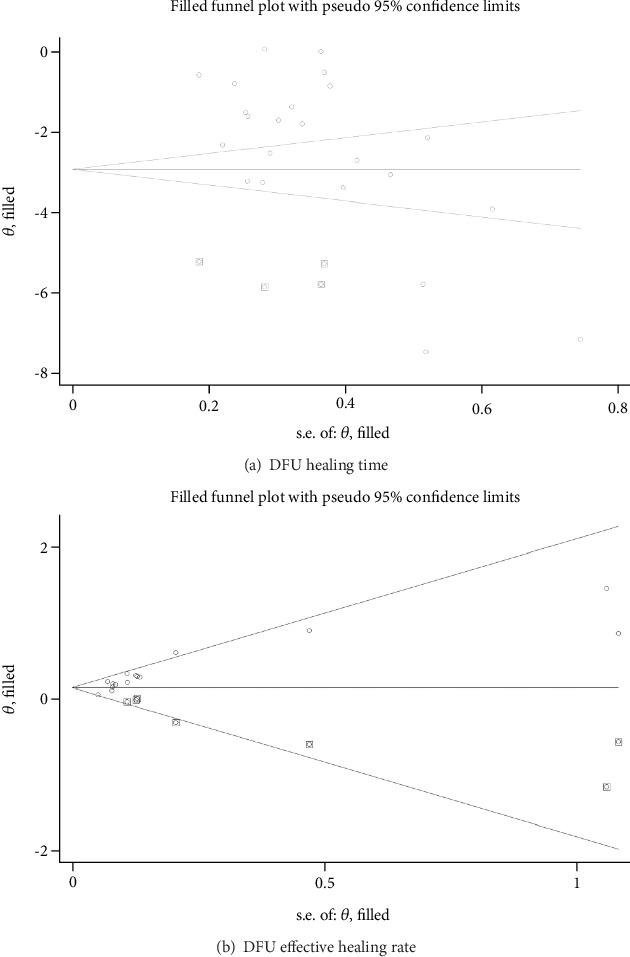
Robustness assessment of ulcer healing time and ulcer healing rate using trim-and-fill analysis. Note: The vertical axis “theta, filled” represents the adjusted estimate of the effect size (*θ*), and the horizontal axis “s.e. of theta, filled” represents the standard error of the adjusted effect size.

To evaluate the robustness of the results, we conducted trim-and-fill analyses. For healing time, four studies were imputed, resulting in increased heterogeneity (*Q* = 1069.112, *p* < 0.001) and a pooled effect size of SMD = −2.922 (95% CI: −3.673 to −2.172), with a more symmetrical funnel plot (Figure 9a). For healing efficiency, seven studies were imputed, yielding a pooled effect size of RR = 1.171 (95% CI: 1.091–1.256) with moderate heterogeneity (*Q* = 36.427, *p* = 0.020). Despite these adjustments, the direction and statistical significance of the results remained consistent (Figure 9b).

### 5.8. Safety Evaluation/Adverse Event Analysis

The potential adverse events associated with the interventions in the included studies were primarily mild systemic discomfort, skin symptoms, and pain. Most studies indicated no allergic reactions or other adverse events between the treatment and control groups [12, 42], or no statistically significant differences in the incidence of adverse events [24, 37, 43]. Additionally, no serious adverse events were reported. Some mild adverse events resolved tablespontaneously after implementing measures such as slowing the administration rate. These findings suggest that the related treatment regimens have favorable safety and tolerability profiles.

## 6. Discussion

### 6.1. Interpretation of Main Findings

This study demonstrates that novel dressings or their combination with traditional dressings significantly outperform traditional dressings alone in reducing wound healing time and improving healing efficiency, aligning with current clinical trends and published evidence [47–53]. The top-performing regimens—such as silver ion dressings combined with basic bFGF or hydrogel and EGF combined with silver ion dressings—leverage mechanisms including antimicrobial activity, fibroblast proliferation modulation, and maintenance of a moist microenvironment [47–50]. Furthermore, the synergistic effects observed in amniotic membrane and PRP combined with hydrogel underscore the advantage of multitarget interventions in wound healing [52, 53].

Despite the superior efficacy of novel dressings, their clinical adoption is hindered by cost, operational complexity, and regional healthcare resource disparities. Traditional dressings remain dominant in primary care due to affordability and ease of use, emphasizing the need to balance efficacy, accessibility, and patient economic burden in clinical decision-making. Additionally, strict glycemic control, debridement, and aseptic management remain foundational to optimizing outcomes, highlighting the importance of integrated therapeutic strategies.

Our findings demonstrate that silver ion dressings combined with bFGF significantly shorten wound healing time, and that combinations involving EGF, amniotic membrane, or PRP with hydrogel markedly improve healing efficiency. When translating these promising outcomes into clinical application, cost and accessibility are critical. Silver ion dressings with bFGF- and EGF-based regimens, although effective, typically incur higher costs than standard dressings—a concern in resource-limited settings. In contrast, more general hydrogel dressings, which our results also highlight favorably, have been reported to be more effective than basic contact dressings (RR 1.80; 95% CI 1.27–2.56) [54] and are relatively affordable and simple to implement. Moreover, PRP gel, akin to our PRP–hydrogel combinations, has been shown to lower long-term care costs for nonhealing DFUs, making it a cost-effective option [55]. Taken together, these observations suggest that while advanced biomaterial combinations offer superior efficacy, hydrogel-based or PRP-enhanced treatments may present a more economically viable and scalable choice for broader adoption in real-world, resource-constrained environments.

### 6.2. Methodological Considerations and Limitations

Several methodological issues and limitations should be acknowledged. The included interventional RCTs varied in methodological rigor, with some lacking strict randomization, blinding, or adequate sample size estimation, potentially compromising internal validity and introducing selection or measurement biases.

Additionally, inconsistencies in intervention definitions, implementation details, and follow-up durations across studies increased heterogeneity, possibly due to variations in dressing application, change frequency, and treatment duration, which may affect the accuracy of efficacy assessments. A key limitation is the heterogeneity in “routine debridement” procedures—such as differences in performers (nurses vs. physicians), tools (sharp/surgical vs. enzymatic/autolytic), and frequency—which were not fully standardized or detailed across studies.

Due to insufficient reporting, we assumed equivalence for analysis, but this could introduce unmeasured variability, potentially biasing effect estimates and limiting the generalizability of our findings. Furthermore, the relatively limited sample size in some comparisons reduces statistical power and increases the risk of false-negative results.

The risk of bias in allocation concealment and blinding also deserves particular attention. Only five of the included RCTs explicitly reported adequate allocation concealment, and blinding was variably implemented, with most studies adopting open-label designs. These methodological limitations raise concerns about potential selection and performance bias. To evaluate the influence of study quality, we performed sensitivity analyses excluding high-risk studies in these domains. The results demonstrated that the overall network remained consistent, as shown by nonsignificant global and local inconsistency tests (*p* = 0.723 for ulcer healing time; *p* = 0.8756 for healing efficiency). However, the ranking of some interventions changed: While PRP gel appeared highly effective in the primary analysis, its superiority was attenuated after excluding high-risk studies, suggesting that its benefit may have been overestimated. Conversely, honey dressings became more competitive under the low-risk scenario, indicating that their relative efficacy may have been underestimated previously. Notably, silver-based dressings consistently ranked among the top interventions across all analyses, underscoring the robustness of this finding. Taken together, these observations highlight that although the principal conclusions of our NMA remain stable, treatment rankings for certain dressings are sensitive to methodological rigor, and the interpretation of comparative efficacy should be made with caution. Future well-designed, adequately blinded RCTs with rigorous allocation concealment are warranted to further validate these findings.

In addition, the asymmetry of the funnel plot and the results of Egger's test suggested potential publication bias and small-study effects. Nevertheless, trim-and-fill sensitivity analyses demonstrated that the overall direction and statistical significance of the results remained unchanged, supporting the robustness of our conclusions.

Moving forward, clinicians should tailor dressing selection to individual patient needs to optimize outcomes, while ongoing advancements in biomaterials and tissue engineering—such as genetically engineered dressings, nanomaterial-based dressings, and smart dressings with controlled growth factor release—offer new opportunities to enhance clinical practice and improve patients' quality of life.

## 7. Conclusion

In conclusion, this NMA highlights the superior efficacy of novel dressings and their combinations with traditional dressings in improving wound healing time and efficiency compared to traditional dressings alone. These findings align with current clinical trends and underscore the potential of advanced dressings, such as silver ion dressings, growth factor–enhanced formulations, and hydrogel-based therapies, to optimize wound care outcomes. However, the broader adoption of these innovative approaches is currently limited by factors such as cost, operational complexity, and regional healthcare disparities. Moreover, sensitivity analyses indicated that conclusions regarding healing time are sensitive to study quality, suggesting that these results should be interpreted with caution. Future research should focus on large-scale, high-quality RCTs to validate these results and explore emerging technologies, including genetically engineered and nanomaterial-based dressings. By integrating evidence-based practices with patient-centered care, clinicians can enhance therapeutic outcomes and improve the quality of life for individuals with chronic or complex wounds.

## Figures and Tables

**Table 1:** PubMed database search strategy.

**SET**	**Search strategy**
#1	(“Diabetic Foot”[Mesh] OR “Diabetic Foot Ulcer”[Mesh] OR “Foot Ulcer”[Mesh] OR “diabetic foot”[Title/Abstract] OR “diabetic foot ulcer”[Title/Abstract] OR “diabetic foot infection”[Title/Abstract] OR “foot ulcer”[Title/Abstract])
#2	(“Biomaterials”[Mesh] OR “Tissue Engineering”[Mesh] OR “Regenerative Medicine”[Mesh] OR “Skin, Artificial”[Mesh] OR “biological material∗”[Title/Abstract] OR “biomaterials”[Title/Abstract] OR “biological dressing∗”[Title/Abstract] OR “tissue engineering”[Title/Abstract] OR “regenerative medicine”[Title/Abstract] OR “growth factor∗”[Title/Abstract] OR “collagen”[Title/Abstract] OR “amniotic membrane”[Title/Abstract] OR “stem cell therapy”[Title/Abstract] OR “hydrogel∗”[Title/Abstract] OR “biological graft∗”[Title/Abstract] OR “scaffold∗”[Title/Abstract])
#3	(“Efficacy”[Title/Abstract] OR “Safety”[Title/Abstract] OR “Treatment Outcome”[Mesh] OR “treatment outcome∗”[Title/Abstract] OR “therapeutic effectiveness”[Title/Abstract] OR “clinical outcomes”[Title/Abstract] OR “risk assessment”[Title/Abstract] OR “adverse effect∗”[Title/Abstract] OR “complications”[Title/Abstract] OR “patient safety”[Title/Abstract])
#4	#1 and #2 and #3

**Table 2:** Basic characteristics of the included literature.

**First author**	**Years**	**Random methods**	**N**	**Age (** **x̅**±**s)**		**Interventions**		**Duration**	**Wagner**	**Outcomes**
			**T/C**	**T**	**C**	**T**	**C**			
Zheng [12]	2007	Random	16/16	44.6 ± 4.8	47.4 ± 5.2	*A* + *G*	*A*	—	—	①
Xu [13]	2009	Random	21/20	—		*A* + *G*	*A*	15 days	—	①
Mayila [14]	2012	Random	20/20	—		*E* + *A*	*A*	—	1~2	②
Manalelsayedez [15]	2012	Random	20/20	39.28 ± 6.27	36.6 ± 9.34	*A* + *F*	*A* + *L*	—	2	①
Kamaratos [16]	2012	Random	32/31	56 ± 14	57 ± 15	*A* + *F*	*A*	16 weeks	1~2	①
He [17]	2014	Random number table methods	25/25	52.3 ± 11.6	51.6 ± 10.7	*A* + *G*	*A*	—	1~2	①
Huang [18]	2015	Random	60/60	48.3 ± 12.6		*A* + *D*	*A*			①
He [19]	2016	Computerized random	40/40	55.7 ± 21.2	58.8 ± 21.9	*A* + *D*	*A*	30 days	—	①②
Liu [20]	2016	Random number table methods	30/30	54.6 ± 9.62	55.4 ± 8.19	*A* + *C*	*A* + *G*	8 weeks	2~3	①
Li [21]	2016	Random	24/24	73.47 ± 2.87	74.35 ± 2.68	*A* + *G*	*A*	—	1~2	①
Mohajeri-Tehrani [22]	2016	Simple random	27/30	55.44 ± 11.27	60 ± 9.3	*A* + *E*	*A*	6 weeks	2~4	②
Sun [23]	2017	Random number table methods	50/50	59.6 ± 4.8	58.7 ± 4.5	*A* + *B* + *D*	*A*	8 weeks	2~3	②
Yang [24]	2017	Random number table methods	38/38	40.1 ± 10.2	43.7 ± 9.8	*A* + *C*	*A*	1 month	3~4	①
Liu [25]	2017	Random	70/70	48.23 ± 6.55	48.02 ± 6.12	*A* + *D*	*A*	—	—	①②
Wu [26]	2017	Random	69/69	—		*A* + *D*	*A*	—	1~3	①②
Zhong [27]	2018	Random	30/30	65.15 ± 5.36	65.10 ± 5.34	*A* + *G*	*A*	—	—	①②
Fu [28]	2018	**Cluster randomization**	32/32	59.87 ± 8.12	58.25 ± 7.94	*A* + *C*	*A* + *B*	8 weeks	2~3	①
Pei [29]	2018	Random number table methods	60/60	52.7 ± 2.6	52.1 ± 2.7	*A* + *D*	*A*	3 months	1~2	①
Singh [30]	2018	Random	29/26	53.76 ± 10.38	55.69 ± 10.35	*A* + *C*	*A*	4 weeks	—	①
Al Saeed [31]	2019	Random	36/35	Male: 67.6 ± 12.7 Female: 63.7 ± 13.4	Male: 69.3 ± 11.8 Female: 62.6 ± 14.1	*A* + *F*	*A* + *D*	—	2~4	①
Ma [32]	2019	Random number table methods	40/40	56.93 ± 2.08	56.52 ± 2.11	*A* + *L* + *D*	*A*	—	—	①
Guo [33]	2019	Random number table methods	27/28	52.3 ± 2.6		*A* + *B* + *D*	*A* + *D*	—	—	②
Xie [34]	2020	Random	30/30	57.41 ± 6.98	56.57 ± 7.24	*A* + *D* + *L*	*A* + *L*	30 days	2~4	①②
Wang [35]	2020	Random number table methods	27/27	61.54 ± 8.06	62.48 ± 7.76	*A* + *C*	*A*	20 days	2~4	①②
Li [36]	2020	Random	43/43	64.86 ± 8.83	63.4 ± 9.52	*A* + *B* + *D*	*A* + *D*	28 days	—	②
Li [37]	2020	Random number table methods	22/23	58.25 ± 3.56	56.57 ± 7.24	*A* + *B*	*A*	14 days	1~3	①②
Chen [38]	2020	Random number table methods	43/43	69.31 ± 9.12	70.82 ± 8.19	*A* + *D*	*A*	8 weeks	2~4	①②
Viswanathan [39]	2020	Random	27/23	57.9 ± 9.6	55.0 ± 6.8	*A* + *B*	*A*	30 days	1~2	①
Elsaid [40]	2020	Computerized random	12/12	54.7 ± 6.6	55.6 ± 6.5	*A* + *C*	*A*	20 weeks	—	①
Game [41]	2021	Online randomization system	15/16	62.8 ± 9.21	57 ± 10.39	*A* + *E*	*A*	12 weeks	—	②
Oliveira [42]	2021	Computerized random (Biostat 5.0)	14/11	60.6 ± 8.6	65.1 ± 6.5	*A* + *B*	*A*	12 weeks	—	②
Hu [43]	2022	Random number table methods	60/60	57.49 ± 9.83	56.82 ± 9.37	*A* + *C*	*A*	10 weeks	2~4	①②
Ren [44]	2024	Random number table methods	30/30	65.31 ± 5.72	65.28 ± 5.37	*A* + *G* + *D*	*A* + *D*	—	2~4	①
Searan [45]	2024	Computerized random	90/30	—		1: *A* + *F* 2: *A* + *L* 3: *A* + *F* + *L*	*A*	—	2	①
Shang [46]	2024	Random	39/39	59.04 ± 8.03	58.77 ± 7.89	*A* + *C* + *L*	*A*	—	2~4	①②

*Note:* Interventions: *A*, traditional dressings (oil sand, cotton cloth, dry gauze, and bandages); *B*, epidermal growth factor; *C*, platelet-rich plasma gel (APG); *D*, antimicrobial dressings (silver ion–based); *E*, biomaterials (amniotic membrane); *F*, honey dressings; *G*, basic fibroblast growth factor; *H*, hydrogel. Outcome indicators: ① wound healing time; ② overall treatment efficacy rate. Cluster randomization: A method of random allocation in which groups (clusters) of participants, rather than individuals, are randomly assigned to different study arms. This approach is commonly used when interventions are delivered at the group level or when contamination between individuals in the same group is likely.

## Data Availability

The data that support the findings of this study are available from the corresponding author upon reasonable request.

## Supporting Information

Supporting Information Additional supporting information can be found online in the Supporting Information section. The supporting figures illustrate the findings from the sensitivity analyses. Figure S1 presents the results of the network meta-analysis comparing different wound dressings for the treatment of diabetic foot ulcers after excluding high-risk studies. Figure S2 shows the SUCRA ranking plot derived from the sensitivity analyses, indicating the relative ranking probabilities of each intervention. Table S1 presents the search strategy of different database.
