# Supplementary material for: Efficacy and Safety of Biomaterials and Antimicrobial Dressings in the Treatment of Diabetic Foot Ulcers: A Systematic Review and Network Meta-Analysis
Source: J Diabetes Res. 2025 Oct 8;2025:1548984. doi: 10.1155/jdr/1548984 (PMC12527605; doi:10.1155/jdr/1548984)
Supplement: Supporting Information — Additional supporting information can be found online in the Supporting Information section. The supporting figures illustrate the findings from the sensitivity analyses. Figure S1 presents the results of the network meta-analysis comparing different wound dressings for the treatment of diabetic foot ulcers after excluding high-risk studies. Figure S2 shows the SUCRA ranking plot derived from the sensitivity analyses, indicating the relative ranking probabilities of each intervention. Table S1 presents the search strategy of different database. [file 1548984.f1.docx]

**Contents**

[*Supplement 1 Detailed search strategy：* 1](#_Toc10865)

[*Supplement 2 Sensitivity Analysis Results After Excluding High-Risk Studies* 8](#_Toc3734)

# *Supplement 1 Detailed search strategy：*

| **Table S1 Search Strategy of different database** | | | |
| --- | --- | --- | --- |
| Database | Set | Search Strategy | Articles |
| PubMed | #1 | ("Diabetic Foot"[Mesh] OR "Diabetic Foot Ulcer"[Mesh] OR "Foot Ulcer"[Mesh] OR "diabetic foot"[Title/Abstract] OR "diabetic foot ulcer"[Title/Abstract] OR "diabetic foot infection"[Title/Abstract] OR "foot ulcer"[Title/Abstract]) | 704 |
|  | #2 | ("Biomaterials"[Mesh] OR "Tissue Engineering"[Mesh] OR "Regenerative Medicine"[Mesh] OR "Skin, Artificial"[Mesh] OR "biological material*"[Title/Abstract] OR "biomaterials"[Title/Abstract] OR "biological dressing*"[Title/Abstract] OR "tissue engineering"[Title/Abstract] OR "regenerative medicine"[Title/Abstract] OR "growth factor*"[Title/Abstract] OR "collagen"[Title/Abstract] OR "amniotic membrane"[Title/Abstract] OR "stem cell therapy"[Title/Abstract] OR "hydrogel*"[Title/Abstract] OR "biological graft*"[Title/Abstract] OR "scaffold*"[Title/Abstract]) |  |
|  | #3 | ("Efficacy"[Title/Abstract] OR "Safety"[Title/Abstract] OR "Treatment Outcome"[Mesh] OR "treatment outcome*"[Title/Abstract] OR "therapeutic effectiveness"[Title/Abstract] OR "clinical outcomes"[Title/Abstract] OR "risk assessment"[Title/Abstract] OR "adverse effect*"[Title/Abstract] OR "complications"[Title/Abstract] OR "patient safety"[Title/Abstract]) |  |
|  | #4 | #1 and #2 and #3 |  |
| Embase | #1 | ('diabetic foot'/exp OR 'diabetic foot ulcer'/exp OR 'foot ulcer'/exp OR 'diabetic foot':ab,ti OR 'diabetic foot ulcer':ab,ti OR 'diabetic foot infection':ab,ti OR 'foot ulcer':ab,ti) | 820 |
|  | #2 | ('biomaterial'/exp OR 'tissue engineering'/exp OR 'regenerative medicine'/exp OR 'artificial skin'/exp OR biomaterial*:ab,ti OR 'antimicrobial dressing*':ab,ti OR 'tissue engineering':ab,ti OR 'regenerative medicine':ab,ti OR 'growth factor*':ab,ti OR collagen:ab,ti OR 'amniotic membrane':ab,ti OR 'stem cell therapy':ab,ti OR hydrogel*:ab,ti OR 'silver ions':ab,ti OR 'biological graft*':ab,ti OR scaffold*:ab,ti) |  |
|  | #3 | (efficacy:ab,ti OR safety:ab,ti OR 'treatment outcome'/exp OR 'treatment outcome*':ab,ti OR 'therapeutic effectiveness':ab,ti OR 'clinical outcomes':ab,ti OR 'risk assessment':ab,ti OR 'adverse effect*':ab,ti OR complication*:ab,ti OR 'patient safety':ab,ti) |  |
|  | #4 | ('randomized controlled trial'/exp OR randomised:ab,ti OR randomized:ab,ti OR 'random allocation':ab,ti OR 'clinical trial'/exp) |  |
|  | #5 | #1 AND #2 AND #3 AND #4 |  |
| Cochrane Library | #1 | ("diabetic foot":ti,ab,kw OR "diabetic foot ulcer":ti,ab,kw OR "diabetic foot infection":ti,ab,kw OR "foot ulcer":ti,ab,kw) | 648 |
|  | #2 | (biomaterial*:ti,ab,kw OR "antimicrobial dressing*":ti,ab,kw OR "tissue engineering":ti,ab,kw OR "regenerative medicine":ti,ab,kw OR "growth factor*":ti,ab,kw OR collagen:ti,ab,kw OR "amniotic membrane":ti,ab,kw OR "stem cell therapy":ti,ab,kw OR hydrogel*:ti,ab,kw OR "silver ions":ti,ab,kw OR "biological graft*":ti,ab,kw OR scaffold*:ti,ab,kw) |  |
|  | #3 | (efficacy:ti,ab,kw OR safety:ti,ab,kw OR "treatment outcome*":ti,ab,kw OR "therapeutic effectiveness":ti,ab,kw OR "clinical outcomes":ti,ab,kw OR "risk assessment":ti,ab,kw OR "adverse effect*":ti,ab,kw OR complication*:ti,ab,kw OR "patient safety":ti,ab,kw) |  |
|  | #4 | (randomised:ti,ab,kw OR randomized:ti,ab,kw OR "random allocation":ti,ab,kw OR "clinical trial":ti,ab,kw) |  |
|  | #5 | #1 AND #2 AND #3 AND #4 |  |
| Web of Science | #1 | TS=("diabetic foot" OR "diabetic foot ulcer" OR "diabetic foot infection" OR "foot ulcer") | 798 |
|  | #2 | TS=(biomaterial* OR "antimicrobial dressing*" OR "tissue engineering" OR "regenerative medicine" OR "growth factor*" OR collagen OR "amniotic membrane" OR "stem cell therapy" OR hydrogel* OR "silver ions" OR "biological graft*" OR scaffold*) |  |
|  | #3 | TS=(efficacy OR safety OR "treatment outcome*" OR "therapeutic effectiveness" OR "clinical outcomes" OR "risk assessment" OR "adverse effect*" OR complication* OR "patient safety") |  |
|  | #4 | TS=(randomised OR randomized OR "random allocation" OR "clinical trial") |  |
|  | #5 | #1 AND #2 AND #3 AND #4 |  |
| MEDLINE (Ovid) |  | (Diabetic Foot/ OR Diabetic Foot Ulcer/ OR Foot Ulcer/ OR diabetic foot.ti,ab. OR diabetic foot ulcer.ti,ab. OR foot ulcer.ti,ab. OR diabetic ulcer.ti,ab.) AND ("diabetic foot".ti,ab. OR "diabetic foot ulcer".ti,ab. OR "foot ulcer".ti,ab. OR "diabetic ulcer".ti,ab.) AND (Biomaterials/ OR Tissue Engineering/ OR Regenerative Medicine/ OR Skin, Artificial/ OR biomaterial*.ti,ab. OR antimicrobial dressing.ti,ab. OR silver ions.ti,ab. OR tissue engineering.ti,ab. OR regenerative medicine.ti,ab. OR growth factor.ti,ab. OR collagen.ti,ab. OR amniotic membrane.ti,ab. OR stem cell therapy.ti,ab. OR hydrogel*.ti,ab. OR biological graft*.ti,ab. OR scaffold*.ti,ab.) AND ("biomaterial*".ti,ab. OR "antimicrobial dressing".ti,ab. OR "silver ions".ti,ab. OR "tissue engineering".ti,ab. OR "regenerative medicine".ti,ab. OR "growth factor*".ti,ab. OR "collagen".ti,ab. OR "amniotic membrane".ti,ab. OR "stem cell therapy".ti,ab. OR "hydrogel*".ti,ab. OR "biological graft".ti,ab. OR "scaffold*".ti,ab.) AND (efficacy.ti,ab. OR safety.ti,ab. OR Treatment Outcome/ OR treatment outcome*.ti,ab. OR clinical outcomes.ti,ab. OR adverse effect*.ti,ab. OR complication*.ti,ab. OR patient safety.ti,ab.) AND ("efficacy".ti,ab. OR "safety".ti,ab. OR "treatment outcome*".ti,ab. OR "clinical outcomes".ti,ab. OR "adverse effect*".ti,ab. OR "complication*".ti,ab. OR "patient safety".ti,ab.) AND (Randomized Controlled Trial.pt. OR Randomized Controlled Trials as Topic/ OR randomised.ti,ab. OR randomized.ti,ab. OR random allocation.ti,ab. OR clinical trial.ti,ab.) AND ("randomised".ti,ab. OR "randomized".ti,ab. OR "random allocation".ti,ab. OR "clinical trial".ti,ab.) | 359 |
| Scopus |  | (TITLE-ABS-KEY (diabetic foot + diabetic foot ulcer + foot ulcer + diabetic ulcer)) AND (TITLE-ABS-KEY ("diabetic foot" + "diabetic foot ulcer" + "foot ulcer" + "diabetic ulcer")) AND (TITLE-ABS-KEY (biomaterial* + antimicrobial dressing* + silver ions + tissue engineering + regenerative medicine + growth factor* + collagen + amniotic membrane + stem cell therapy + hydrogel* + biological graft* + scaffold*)) AND (TITLE-ABS-KEY ("biomaterial*" + "antimicrobial dressing"* + "silver ions" + "tissue engineering" + "regenerative medicine" + "growth factor*" + "collagen" + "amniotic membrane" + "stem cell therapy" + "hydrogel*" + "biological graft*" + "scaffold*")) AND (TITLE-ABS-KEY (efficacy + safety + treatment outcome* + clinical outcomes + adverse effect* + complication* + patient safety)) AND (TITLE-ABS-KEY ("efficacy" + "safety" + "treatment outcome*" + "clinical outcomes" + "adverse effect*" + "complication*" + "patient safety")) AND (TITLE-ABS-KEY (randomised + randomized + random allocation + clinical trial)) AND (TITLE-ABS-KEY ("randomised" + "randomized" + "random allocation" + "clinical trial")) | 1755 |
| CNKI | #1 | (SU='糖尿病足' OR SU='糖尿病足溃疡' OR SU='足溃疡' OR KY='糖尿病足' OR KY='糖尿病足溃疡' OR KY='糖尿病足感染' OR KY='足溃疡') | 368 |
|  | #2 | (SU='生物材料' OR SU='抗菌敷料' OR SU='组织工程' OR SU='再生医学' OR KY='生物材料' OR KY='抗菌敷料' OR KY='银离子敷料' OR KY='生长因子' OR KY='富血小板血浆' OR KY='羊膜' OR KY='水凝胶' OR KY='蜂蜜敷料' OR KY='组织工程' OR KY='再生医学' OR KY='生物移植物' OR KY='支架') |  |
|  | #3 | (KY='疗效' OR KY='安全性' OR KY='治疗效果' OR KY='愈合时间' OR KY='不良反应' OR KY='并发症') |  |
|  | #4 | (KY='随机对照试验' OR KY='随机分配' OR KY='临床试验') |  |
|  | #5 | #1 AND #2 AND #3 AND #4 |  |
| VIP | #1 | (M='糖尿病足' OR M='糖尿病足溃疡' OR M='足溃疡' OR K='糖尿病足' OR K='糖尿病足溃疡' OR K='糖尿病足感染' OR K='足溃疡') | 278 |
|  | #2 | (M='生物材料' OR M='抗菌敷料' OR M='组织工程' OR M='再生医学' OR K='生物材料' OR K='抗菌敷料' OR K='银离子敷料' OR K='生长因子' OR K='富血小板血浆' OR K='羊膜' OR K='水凝胶' OR K='蜂蜜敷料' OR K='组织工程' OR K='再生医学' OR K='生物移植物' OR K='支架') |  |
|  | #3 | (K='疗效' OR K='安全性' OR K='治疗效果' OR K='愈合时间' OR K='不良反应' OR K='并发症') |  |
|  | #4 | (K='随机对照试验' OR K='随机分配' OR K='临床试验') |  |
|  | #5 | #1 AND #2 AND #3 AND #4 |  |
| Wanfang Data | #1 | (主题:糖尿病足 OR 主题:糖尿病足溃疡 OR 主题:足溃疡 OR 关键词:糖尿病足 OR 关键词:糖尿病足溃疡 OR 关键词:糖尿病足感染 OR 关键词:足溃疡) | 188 |
|  | #2 | (主题:生物材料 OR 主题:抗菌敷料 OR 主题:组织工程 OR 主题:再生医学 OR 关键词:生物材料 OR 关键词:抗菌敷料 OR 关键词:银离子敷料 OR 关键词:生长因子 OR 关键词:富血小板血浆 OR 关键词:羊膜 OR 关键词:水凝胶 OR 关键词:蜂蜜敷料 OR 关键词:组织工程 OR 关键词:再生医学 OR 关键词:生物移植物 OR 关键词:支架) |  |
|  | #3 | (关键词:疗效 OR 关键词:安全性 OR 关键词:治疗效果 OR 关键词:愈合时间 OR 关键词:不良反应 OR 关键词:并发症) |  |
|  | #4 | (关键词:随机对照试验 OR 关键词:随机分配 OR 关键词:临床试验) |  |
|  | #5 | #1 AND #2 AND #3 AND #4 |  |
| CBM | #1 | ('糖尿病足' OR '糖尿病足溃疡' OR '足溃疡' OR '糖尿病足感染') | 235 |
|  | #2 | ('生物材料' OR '抗菌敷料' OR '银离子敷料' OR '生长因子' OR '富血小板血浆' OR '羊膜' OR '水凝胶' OR '蜂蜜敷料' OR '组织工程' OR '再生医学' OR '生物移植物' OR '支架') |  |
|  | #3 | ('疗效' OR '安全性' OR '治疗效果' OR '愈合时间' OR '不良反应' OR '并发症') |  |
|  | #4 | ('随机对照试验' OR '随机分配' OR '临床试验') |  |
|  | #5 | #1 AND #2 AND #3 AND #4 |  |

| **Table S2 Summary of literature identification, screening, and inclusion** | | |
| --- | --- | --- |
| **Screening Stage** | **Reason for Exclusion** | **Number of Articles** |
| **Initial Records** | Identified through database searches | 6,153 |
| **Duplicates Removed** | Duplicate references | 2,715 |
| **Title & Abstract Screening** | Reviews and meta-analyses | 850 |
|  | Conference abstracts and dissertations | 720 |
|  | Cell or in vitro experiments | 610 |
|  | Studies unrelated to diabetic foot ulcers | 510 |
|  | Studies with mismatched population or intervention | 311 |
| **Full-Text Assessment** | Non-randomized controlled studies | 115 |
|  | Articles without results | 31 |
|  | Animal experiment studies | 198 |
|  | Non-journal publications | 58 |
| **Included in Qualitative Synthesis** | Eligible studies | 35 |
| **Final articles included** | **35** | |

# *Supplement 2 Sensitivity Analysis Results After Excluding High-Risk Studies*

## 1.Inconsistency Test Results

For the outcome of ulcer healing time, after excluding high-risk studies, the global inconsistency test yielded *P* = 0.723. Node-splitting analyses for local inconsistency showed that all pairwise comparisons had *P* > 0.05. For the outcome of ulcer healing efficiency, the global inconsistency test yielded *P* = 0.8756, and node-splitting analyses similarly indicated no significant local inconsistency (all *P* > 0.05). The 95% confidence intervals of the inconsistency factors (IF) for both outcomes included 0.

## 2.Ulcer Healing Time

### 2.1 Network Meta-Analysis under the Consistency Model

Traditional dressings combined with silver ion dressings and hydrogel, traditional dressings combined with silver ion dressings and basic fibroblast growth factor, traditional dressings combined with silver ion dressings alone, traditional dressings combined with platelet-rich plasma, and traditional dressings combined with honey dressings were all superior to traditional dressings alone (*P* < 0.05). No significant differences were observed among the other interventions (*P* > 0.05) (***Fig. S1a).***

### 2.2 Cumulative Probability Ranking Results of Included Studies

According to the SUCRA values, the ranking of dressing methods was as follows:A+D+L（80.9%）>A+G+D（79.5%）>A+F（68.8%）>A+D（64.4%）>A+L（59.1%）>A+C（57.8%）>A+C+L（30.4%）>A+G（30.0%）>A+B（23.7%）>A（5.4%）***(Fig. S2a).***

In the primary analysis, the top four interventions were silver combined with basic fibroblast growth factor (bFGF), silver combined with hydrogel, silver alone, and platelet-rich plasma (PRP) gel. After excluding high-risk studies, the ranking changed: silver combined with hydrogel rose to the top position, followed by silver combined with bFGF, honey dressing, and silver alone. These changes suggest that the apparent superiority of PRP gel may largely rely on high-risk studies. Conversely, when the analysis was restricted to lower-risk studies, honey dressing appeared more competitive, indicating that its relative benefit might have been underestimated previously. Overall, silver-based dressings consistently remained within the top four, supporting their robustness across different analytical scenarios.The conclusions may be influenced by study quality, warranting cautious interpretation.

## 3.Healing Efficiency

### 3.1 Network Meta-Analysis under the Consistency Model

Traditional dressings combined with epidermal growth factor, and traditional dressings combined with epidermal growth factor plus silver ion dressings were superior to traditional dressings combined with silver ion dressings alone (*P* < 0.05). Traditional dressings combined with epidermal growth factor, amniotic membrane, platelet-rich plasma plus hydrogel, epidermal growth factor plus silver ion dressings, basic fibroblast growth factor, platelet-rich plasma, or silver ion dressings were all superior to traditional dressings alone. Moreover, traditional dressings combined with epidermal growth factor plus silver ion dressings were superior to traditional dressings combined with silver ion dressings (*P* < 0.05). No significant differences were observed among the other interventions (*P* > 0.05) ***(Fig. S1b)***.

### 3.2 Cumulative Probability Ranking Results of Included Studies

According to the SUCRA values, the ranking of dressing methods was as follows:A+B（93.5%）>A+E（65.4%）>A+C+L（64.3%）>A+B+D（63.5%）>A+G（54.2%）>A+C（33.0%）>A+D（25.7%）>A（0.4%）)***(Fig. S2b)***.

After excluding high-risk studies, the effect estimates remained broadly consistent, suggesting robustness of this finding.


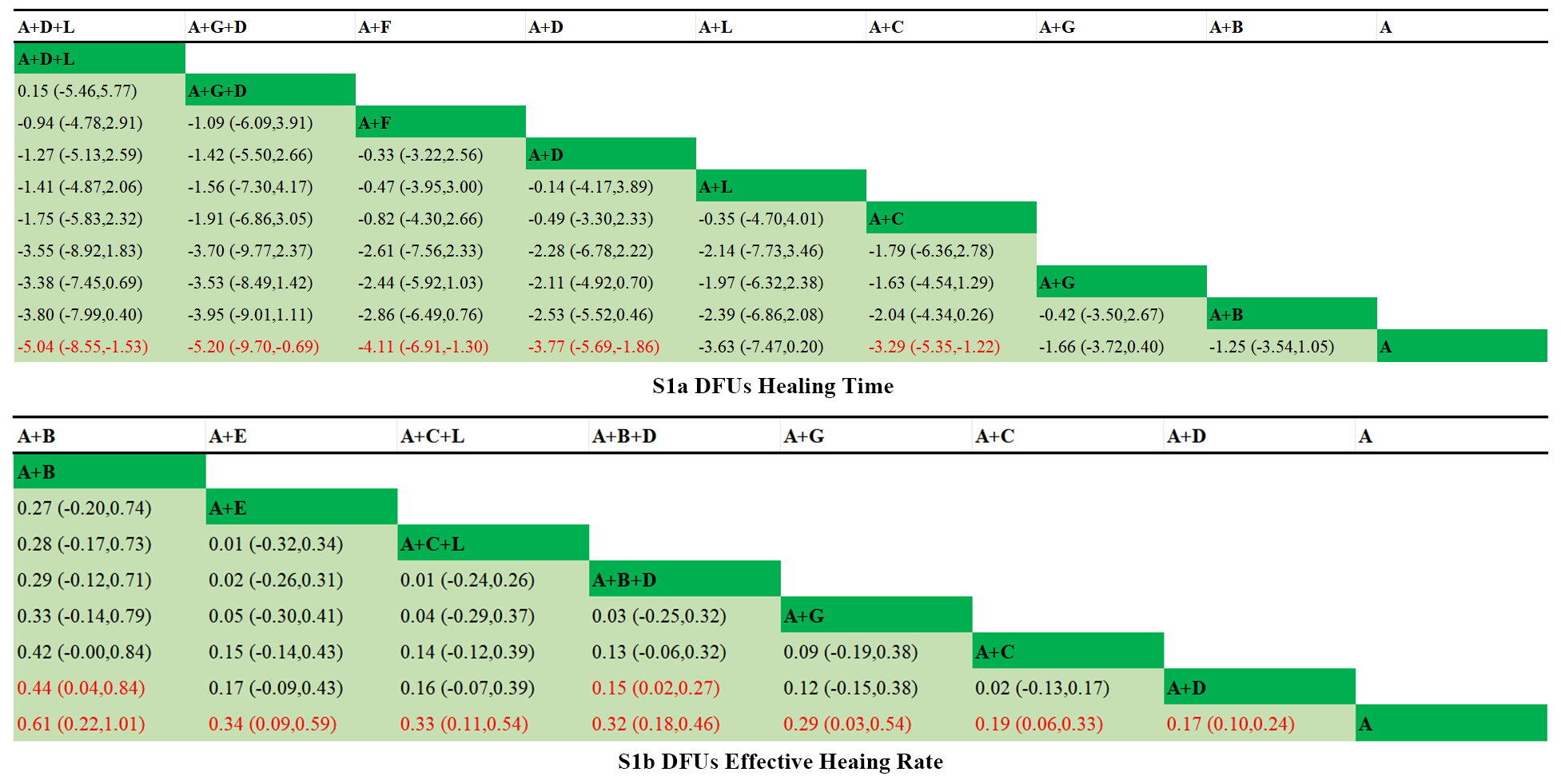


**Fig. S1 Results of Network Meta-analysis for different Wound Dressings in DFUs Treatment** **(after sensitivity analyses)**


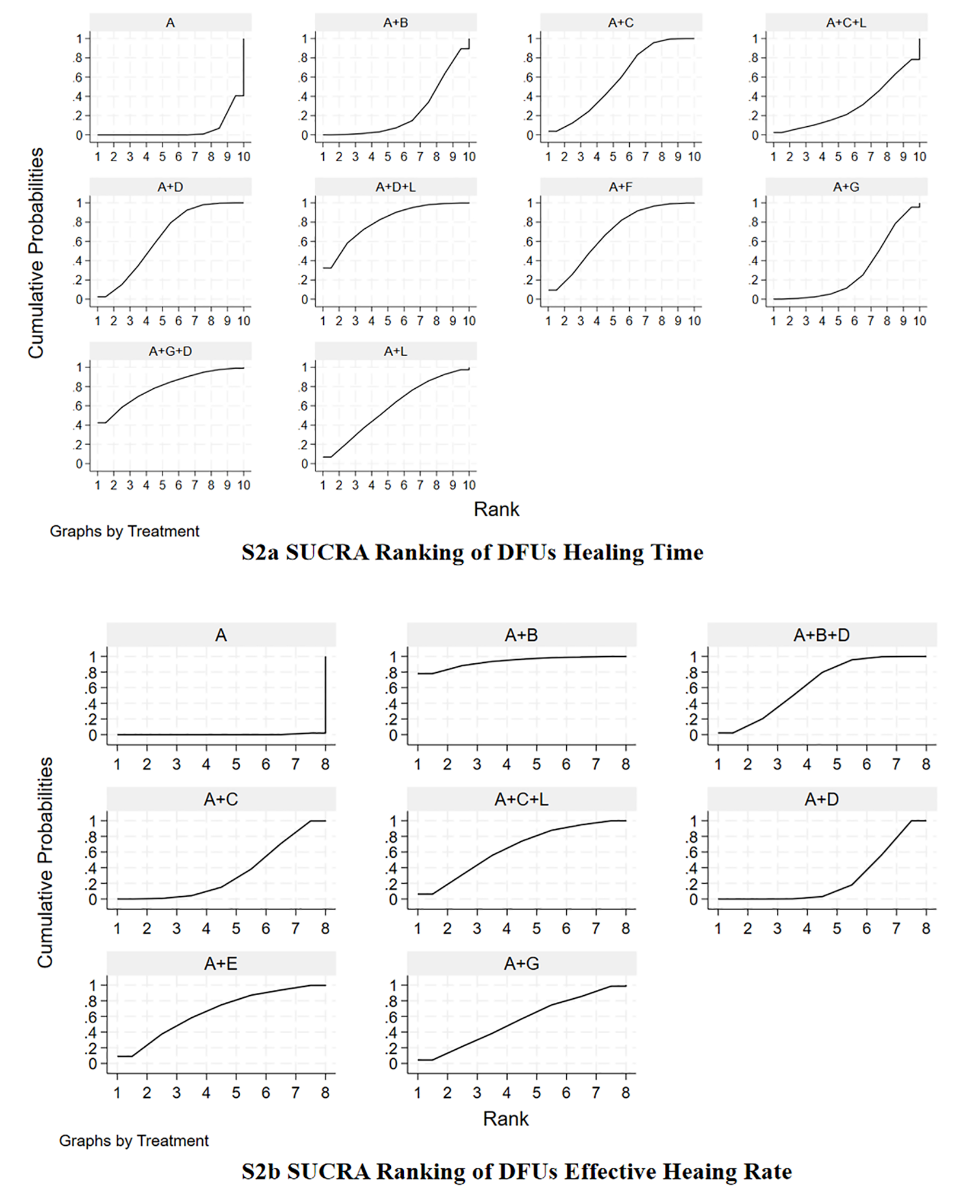


**Fig. S2 SUCRA Ranking Plot (after sensitivity analyses)**
